# Book Notices

**Published:** 1891-08

**Authors:** 


### Book Notices.

ESSENTIALS OF ANATOMY AND MANUAL OF PRACTICAL DISSECTION, together with the Anatomy of the Viscera, prepared especially for students of Medicine, by Chas. B. Nancrede, M. D., professor of surgery and clinical surgery in the University of Michigan, Ann Arbor. Third edition, revised and enlarged, based upon the last edition of Gray. Thirty handsome full-page lithographic plates, in colors, and 180 fine woodcuts. W. B. Saunders, publisher, Philadelphia, 1890, pp. 388, price $2.00.

---

QUIZ-COMPEND NO. 7—A COMPEND OF GYNÆCOLOGY, by Henry Morris, M. D., Philadelphia, with 45 illustrations. Philadelphia: P. Blakiston, Son & Co., 1012 Walnut street, 1891. Cloth, price $1.00.

This is a compact little book of 175 pages. It is not a compend of questions and answers, although it is called a quiz-compend. It embraces a great deal in a small space, and is intended for a hasty review of the subject. B.

---

PHYSICAL DIAGNOSIS AND PRACTICAL URINALYSIS. — An epitome of the physical signs of the heart, lungs, liver, kidney and spleen in health and disease. By John E. Clark, M. D., professor of general chemistry and physics in the Detroit College of Medicine. Fully illustrated. Issued by the Illustrated Medical Journal Co., Detroit, Mich., pp. 200, price post paid $1.00.

This is a nicely gotten up little book and presents a systematic and condensed course of physical diagnosis and urinalysis. It is suitable for students and practitioners. The part on urinalysis is quite comprehensive and is the best part of the book. B.

---

HEREDITY, HEALTH AND PERSONAL BEAUTY, by John V. Shoemaker, A. M., M. D., Philadelphia. Publisher: F. A. Davis, Philadelphia and London, pp. 422, $3.50; 1890.

Advanced views on heredity as applied to the transmission of both health and morbid condition are of great interest to the physician. Any one wishing to be informed on the subject could not do better than to purchase a copy of the above book from the publisher and read it carefully. The work contains 37 chapters, as follows: The general laws of health; the regulation law of life and growth; nature's evidence of the law of life and growth; man's spiritual place in nature; man's physical place in nature; phenomena of evolution in the present era; the sentiment of the beautiful; the source of the beauty of the fair sex; the effect of environment and training on the physique; grace the crown of beauty, etc., etc. B.

---

SAUNDERS' QUESTION-COMPEND NO. 12—ESSENTIALS OF MINOR SURGERY AND BANDAGING, with an appendix of venereal diseases, arranged in the form of questions and answers, prepared especially for students of medicine. By Edward Martin, A. M., M. D., instructor in operative surgery, University of Pennsylvania, etc. Illustrated. Philadelphia: W. B. Saunders, 913 Walnut street. 1890. Cloth $1.00; pp. 166.

---

THE DAUGHTER, her health, education and wedlock. Homely suggestions for mothers and daughters, by Wm. M. Capp, M. D. F. A. Davis, publisher, Philadelphia and London, 1891; pp. 140.

This is a book that should be read by every mother. It treats of the education of her daughter, and has a special view to the practical in her training and future development. "What is to become of our daughters?" is a live theme in many of the leading magazines at this time. The family physician has a strong second in the teaching of this book. B.

---

TWELVE LECTURES ON THE STRUCTURE OF THE CENTRAL NERVOUS SYSTEM, for Physicians and Students; by Dr. Ludwig Edinger, Frankfort-on-the-Main. Second revised edition, with 133 illustrations. Translated by Willis Hall Vittum, M. D., St. Paul, Minn. Edited by C. Eugene Riggs, A. M., M. D., Professor of Mental and Nervous Diseases, University of Minnesota. F. A. Davis, publisher; Philadelphia and London: 1891. Pages 230; $1.75.

This work brings the anatomy and physiology of the central nervous system well up to date. Since the appearance of the first edition of this book, in 1885, much that is new has been discovered. The chapters on the histology and histogenesis have been entirely re-written, as also the sections on the oculomotor, the acoustic, and the fibres of the deep marrow. The portion of the work devoted to comparative anatomy of the nervous system is especially interesting. The book as a whole can be accepted as a most reliable and trustworthy guide in the investigation and study of this very intricate subject B.

---

TEXT BOOK OF HYGIENE—A Comprehensive Treatise of the Principles and Practice of Preventive Medicine from an American standpoint. By George H. Rohe, M. D., Baltimore. Second edition; Philadelphia, F. A. Davis, 1890.

This book is justly regarded as a standard work. It is well printed, in good, clear type, and presents a neat appearance, as do all publications by F. A. Davis.

The chapters on the Germ Theory of Disease, Contagion and Infection, History of Epidemic Diseases, and on Antiseptics, Disinfectants and Deodorants are especially clear and valuable.

B.

---

"INTESTINAL Diseases of Children;" in 2 volumes; by A. Jacoby, M. D., New York, 1890.

"ELECTRICITY; its Application in Medicine;" in 2 volumes; by Wellington Adams, M. D.; Denver, Col., 1891.

"HYPODERMIC Medication;" by Bourneyville and Bricon, '91.

"PRACTICAL Notes on Urinalysis;" by W. B. Canfield, M. D., Baltimore.

"PRACTICAL Points in the Management of the Diseases of Children;" by I. N. Love, M. D., St. Louis.

"TAKING COLD;" by F. H. Bosworth, M. D., New York.

These little books are published by George S. Davis, Detroit. 25 cents each. Paper cover, and contain about 100 pages each. Physicians' leisure library series.

---

"HARE'S TEXT BOOK on Practical Therapeutics," which was mentioned in the Book Department of the JOURNAL last month, is published by Lea Bros. & Co., Philadelphia,—a fact we omitted to mention in connection with the notice. It is a valuable book, handsomely gotton up.

